# Efficacy and Effectiveness of Universal School-Based Wellbeing Interventions in Australia: A Systematic Review

**DOI:** 10.3390/ijerph20156508

**Published:** 2023-08-02

**Authors:** Harshi Gunawardena, Alexander Voukelatos, Sham Nair, Shane Cross, Ian B. Hickie

**Affiliations:** 1 Brain and Mind Centre, Faculty of Medicine and Health, The University of Sydney, Camperdown 2050, Australia; ian.hickie@sydney.edu.au; 2 Sydney Local Health District, Camperdown 2050, Australia; alexander.voukelatos@health.nsw.gov.au; 3 Curriculum and Reform Directorate, NSW Department of Education, Sydney 2001, Australia; sham.nair@det.nsw.edu.au; 4 Orygen, Parkville 3052, Australia; shane.cross@orygen.org.au; 5 School of Psychology, Faculty of Science, The University of Sydney, Camperdown 2006, Australia

**Keywords:** wellbeing, mental health, school, effectiveness, early intervention

## Abstract

The World Health Organisation defines health in terms of wellbeing, and wellbeing has become both a construct and a measure of impact in early intervention and prevention programs in schools. In Australia, schools report on their wellbeing initiatives and there is a plethora of government-funded wellbeing programs already in place in schools. However, education systems and stakeholders worldwide are facing significant challenges with mixed evaluation results of program impact and intervention effect. To better support students, schools, school-based healthcare workers, and community, it is important to know about the effectiveness of school-based programs; yet in the last decade, there has been no national appraisal of these programs in Australia. This systematic review aims to report on the effectiveness of Australian school-based wellbeing programs through a search of 13 databases. Out of 2888 articles, 29 met inclusion criteria. The results found that seventeen interventions comprising 80% of the total number of participants reported no statistically significant intervention effect on wellbeing outcomes. We argue that supporting wellbeing through robust program intervention is important as wellbeing presents both an indication of later onset of more serious mental health issues, and an opportunity for early intervention to break the trajectory leading to full disorder.

## 1. Introduction

This special issue of the *International Journal of Environmental Research and Public Health* offers the opportunity to problematize the meaning and application of wellbeing within an educational context. The purpose of this paper is to critically analyse the meanings and measures of wellbeing in school-based interventions and to provide an objective appraisal of intervention effect. Previous school-based reviews have reported on wellbeing as a secondary measure to programs whose primary measures were related to mental health and psychiatric disorder. Review of only wellbeing based on the critical analysis of how wellbeing is measured within educational settings is sparse. The purpose of this systematic review is to report on the efficacy and effectiveness of school-based wellbeing interventions that use validated wellbeing instruments to identify an intervention effect on the wellbeing outcomes of school children and adolescents. In so doing, we also aim to problematize the meaning of wellbeing and to establish how it can be measured through validating measuring instruments.

Wellbeing is a popular term that has entered the vernacular throughout the English-speaking world today. However, the meanings and multidimensional nature of wellbeing present a major challenge to researchers and healthcare workers to understand and measure how wellbeing manifests in a child or adolescent’s life. In its broadest reading, wellbeing can be seen as a state in which an individual or group can have access to resources to meet basic needs in terms of social norms (objective wellbeing) and individual experiences (subjective wellbeing) [1,2]. In the latter case, wellbeing may be emotion-based (hedonic wellbeing) or involve a state for optimal functioning (*eudaimonic* wellbeing) [3].

The Organisation for the Economic Co-operation and Development (OECD) identified wellbeing as a goal related to 11 dimensions of life: income and wealth, work and job quality, housing, health, knowledge and skills, environmental quality, subjective wellbeing, safety, work–life balance, social connections, and civil engagement [4,5]. A recent systematic review of 105 articles on the community wellbeing of 12–35-year-olds concluded that none of the papers included a definition of wellbeing [6]. Indeed, their review identified 22 relevant themes specifically related to youth mental health and wellbeing including positive emotions (feel and create pleasant emotions, gratitude, etc.), self-efficacy (strengths, human agency, etc.), life satisfaction (global assessment of one’s life), and personal growth (goal achievement, life aspirations, etc.).

The association of mental health with wellbeing was established by the World Health Organization (WHO) when they defined mental health as “a state of wellbeing” [7]. The WHO definition of mental health involves not only the absence of mental disorder but also positive meaning and life satisfaction. An in-depth analysis of wellbeing and its usage within the Australian context by Powell and Graham suggests that mental health and wellbeing may be aligned in both policy and programs, but there is a lack of clarity around what wellbeing means in relation to mental health and how it may be measured [8]. Moreover, researchers have sought to establish that mental health and wellbeing are not equivalent [9,10] and wellbeing is not featured as a measure in the criteria in the Diagnostic and Statistical Manual of Mental Disorders.

Schools play a significant role in supporting the mental health as well as wellbeing of children and adolescents [11,12,13]. School wellbeing is distinct from global definitions of wellbeing in that is it tied into educational outcomes for children and adolescents. A definition of wellbeing in school educational contexts is provided by Noble and colleagues: “Student wellbeing is … linked to …. satisfaction with life at school, their engagement with learning and their social-emotional behavior” [14]. Within the mental health service delivery model, schools implement Tier 1 to 3 levels of intervention [15]. Tier 1 services are universal preventative interventions based on promoting healthy behaviour and mitigating known risk factors. Most school-based interventions fall into Tier 1. Schools may also deliver a small number of Tier 2 services, which are targeted group interventions for students who have developed or are at risk of developing low-level mental health problems at subclinical level. Tier 3 school-based interventions are individualized programs providing intensive support.

In Australia, wellbeing is increasingly embedded into school practice and is supported by policy initiatives, most recently the Alice Springs (Mparntwe) Education Declaration [16] and the Australian Student Wellbeing Framework [17]. By contrast to previous policy initiatives, these policy documents and frameworks emphasize meeting the individual needs of students and specifically focus on “supporting the wellbeing, mental health and resilience of young people” [16] (Para 4). There are three ways that school-based wellbeing services address this [18]. First, schools provide support staff via the school pastor, tutors, classroom teachers, and school counsellors. Second, schools provide mental health and wellbeing knowledge through the school curriculum and programs. Third, schools provide access to wellbeing events including mindfulness sessions and wellbeing days. Combined, they are believed to form an effective whole of school approach that is supported in Australia through KidsMatter and MidMatter [19] and the suite of programs BeYou [20].

Despite progressive national reforms [21,22,23], however, the Australia Productivity Commission Report found: “The Australian Government’s …. initiatives do not address the fundamental issues that impede schools from making a measurable difference to mental health and wellbeing” [24]. Most wellbeing interventions within school contexts are universal preventative programs and a research report by the Australian Council for Education Research found: “there is little clear evidence about the effectiveness of school-based wellbeing programs in terms of their impact on both students’ wellbeing and on academic outcomes” [25] (p. 3).

Part of the challenge has been to identify the measures that relate to wellbeing: should wellbeing measures include all mental disorders that can be measured through diagnostic instruments; or should wellbeing include all measures related to psychosocial aspects? The OECD recommends measuring wellbeing using standardized and validated wellbeing measuring instruments [26]. These include but are not limited to the ‘Psychological Well-Being Scale’ and ‘Flourishing Scale’, where the latter is used due to the meaning of wellbeing being given as ‘flourishing’ [6]. Other key terms related to wellbeing include ‘life satisfaction’ [27], happiness [28,29,30], and ‘resilience’ [31,32,33]. As mitigating strength-based measures, protective factors and coping skills are associated with the ability to maintain wellbeing [34,35,36]. Recently, self-esteem [37] and self-efficacy [6] have been included as a measure of wellbeing.

There is a scarcity of systematic reviews of universal school-based interventions that address effectiveness in terms of multiple criteria: study quality, relative effect size, and statistical significance of intervention impact. Recently, there has been an increased research output, including systematic reviews and meta-analyses, of school-based mental health and wellbeing. The primary target measure in these reviews is mental health outcomes based on diagnostic instruments, while wellbeing measures are reported as secondary outcomes. An extensive summary of global school-based programs by Berger and colleagues reported that programs with long-term outcomes tended to implement cognitive therapy (CBT), social and emotional skills, and mental health literacy [38]. Other wellbeing reviews suggest that early intervention and participant age is a key factor related to implementation effect [39,40]. On the other hand, a systematic review by Moore and colleagues, which featured no Australian interventions, reported that intervention effect and sustainability is a structural factor related to school governance, rather than being defined by intervention characteristics [41]. However, targeted interventions have a greater statistically significant effect on intervention outcomes than universal interventions [42,43,44]. Given that most wellbeing programs are universal, they may show little or a small statistically significant intervention effect [43]. One multicomponent review of psychological and subjective school wellbeing identified one Australian study [45]. This review showed a small but significant improvement in psychological wellbeing remaining over time. Another multicomponent based on positive psychology also suggests that a higher number of sessions is likely to yield positive results [39]. A review looking at the effect of interventions focusing on positive psychology on subjective wellbeing reported a small effect favouring teacher-delivered interventions [45], although there is debate around whether delivery of mental health programs is more effective when delivered by teachers, program staff, or healthcare workers [46]. That review did not include any Australian studies. Another systematic review examining the impact of interventions on subjective wellbeing found four Australian studies out of 55 and reported that only one third of interventions employed strong experimental designs and that positive results were mainly found in studies with a poor study design [39]. A review of 29 school-based mental health and emotional well-being programs identified only one study that was Australian from within the same search period. The study identified three key program themes: increased help-seeking, mental health literacy, and increased social and emotional wellbeing [47]. The studies showed promising results but suffered from weak study designs. An international review looking at outcomes related to mental health and well-being included 10 Australian studies and concluded that half of the studies in the review showed a positive impact. However, the review was limited to psychological, psychosocial, and subjective wellbeing, and did not provide statistical analyses of effect [36].

Many of the studies in these reviews had flawed research designs which limits the generalizability and validity of their results. In addition, few Australian studies were included in these reviews. Collectively, reviews of wellbeing have provided partial direction for educators and researchers in that wellbeing is often secondary to mental health outcomes, is poorly defined, measured, and lacking clarity in terms of identifying program effectiveness. Australian studies feature minimally or are absent in global systematic reviews of school-based wellbeing programs. First, we aim to identify Australia wellbeing programs and second, we seek to establish effectiveness of programs specifically in terms of wellbeing outcomes as part of a universal intervention and prevention strategy for children and young people. In the present review we found 29 interventions within the Australian context, and our search terms focused on wellbeing outcomes alone (and its connecting measures, such as flourishing and resilience, for example). We also provide statistically measures of outcomes reported on validated measuring tools for the purpose of providing statistical clarity to the efficacy descriptions given in key reviews. The purpose of this systematic review is to address the second part of this statement, which is related to school-based wellbeing outcomes.

## 2. Materials and Methods

### 2.1. Search Strategy

**Figure 1:**
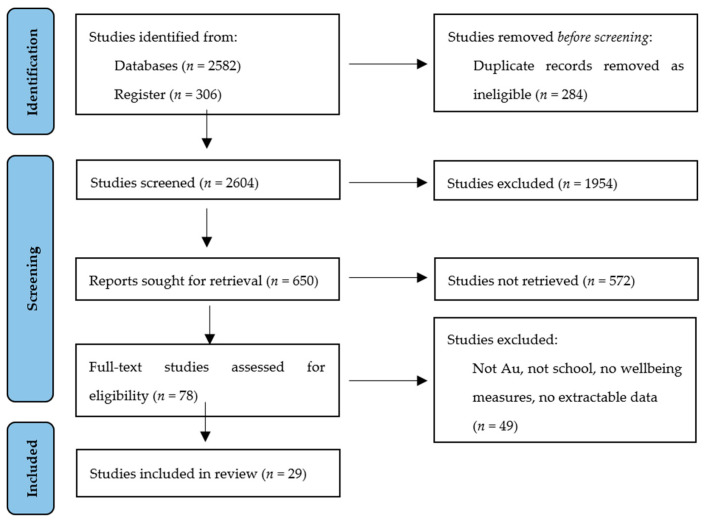
PRISMA flow diagram.

The search strategy in this systematic review used Preferred Reporting Items for Systematic Reviews and Meta-Analyses [48] (Figure 1).

Eleven databases were included in this review (A+Education, BEI, Bibliomap, Embase, Epistemonikos, ERIC, MEDLINE, PsycINFO, PubMed, Scopus, TRoPHI EPPI) and an additional four databases were used for cross-referencing (Campbell Systematic Reviews, Cochrane Central Register of Controlled Trials, Dissertations and Theses via Proquest, and DoPHER Database of Promoting Health Effectiveness Reviews). These were supplemented with internet searches on www.googlescholar.com, www.scirus.com, and www.alta-vista.com (accessed on 2 January 2023). The search strategy for the database searches is given in Table 1.

Duplicates were removed and two authors (HG and AV) independently read the titles and abstracts. Full-text articles were then screened for eligibility. A third reviewer (SC) was used to resolve disagreement regarding eligibility. In total, 2298 results were obtained in the first database search.

### 2.2. Eligibility Criteria

Articles eligible for inclusion were school-based interventions that measured the impact on the wellbeing of young people and adolescents. These are measured through validating measuring instruments for measuring wellbeing, flourishing or *eudaimonia* as outlined by the OECD [26]. A broad reading of wellbeing was taken that includes happiness and resilience measures [12,49]. Studies were included that provided an effect for time and condition on pre- and post-intervention measures and a control group, either active, placebo, or waitlist [50]. If the intervention occurred outside of school grounds, such as nature walks or a sporting activity, then the study was included if it was organized through the school in terms of recruitment of participants and obtaining consent [38]. Articles were filtered for the English language and published between 2012 and 2022.

Studies that did not report on wellbeing outcomes were excluded. This review excluded unpublished doctoral theses, conference material, and articles without empirical data including letters, commentaries, memorandums, and opinion pieces, as they have not undergone a peer-review process. For programs that involved multiple publications, the first study published during the review period was taken [37]. For interventions with a non-school component, such as a pre-school/kindergarten component, only the school-component was taken insofar as the school data could be extracted. There are many mental health-based programs currently running in Australian schools [38,51] that did not meet the inclusion criteria for systematic review.

### 2.3. Data Extraction and Analysis

In line with inclusion and exclusion criteria, data extraction was carried out using the PICOTS method [52]. Records were listed in an Excel sheet under the following categories: authors; year of publication; program name; population (age, mental health condition); intervention (study quality, delivery personnel, exposure, follow-up); outcome (intervention effect (all mental health outcomes, effect size, and assessment instrument used to measure effect); timing; and setting (universal school-based context or school-based external context, e.g., camp). Extraction of data related to intervention characteristics specific to wellbeing included: general wellbeing, emotional wellbeing, psychosocial wellbeing, social wellbeing, subjective wellbeing, coping styles, flourishing, life satisfaction, quality of life, protective factors, resilience, self-esteem, self-efficacy.

Data were extracted by two reviewers, double-blinded by listing who conducted blinded reviews of articles based on title and abstract search (HG and AV). The percentage of the coding decisions on which pairs of coders agreed was used to determine inter-coder reliability and was calculated as 90%. Differences were resolved by a third reviewer (SC).

### 2.4. Risk of Bias

The studies were appraised using the Effective Public Health Practice Project (EPHPP) quality assessment tool [53]. This tool has previously been used to assess quality of wellbeing programs generally [54], and in one of the key systematic reviews of randomized and non-randomised trials in Australia [42]. Assessment criteria were: selection bias, study design, confounding, blinding, data collection, and study attrition. Each criterion was rated 0, 1, or 2 where 2 was given for high quality. The maximum score is 12. A low, medium, and high study quality score refer to the range of 1–4, 5–8, and 9–12, respectively. We felt this method was historical appropriate to the Australian context and it assesses bias in all studies under the same criteria, which is significant given the high number of quasi-experimental studies related to wellbeing in this review.

Each article was independently assessed for quality by two reviewers (HG and AV). Discrepancies were resolved using a third author, SC.

### 2.5. Effect Size

Where data were available and extractable, effect size was calculated to obtain statistically significant effects for time and condition impact on pre- and post-intervention measures. Cohen’s *d* was calculated using the difference between estimated means of the two conditions (intervention and control over time) divided by the baseline standard deviation of raw scores [55]. The range for Cohen’s *d* was: 0.2, 0.4, and 0.7, for small, medium, and strong effect, respectively [56]. Where data were not able to be converted to Cohen’s *d*, the effect size was reported verbatim as authors reported on the manuscript.

## 3. Results

Out of 2298 records, 29 (*N* = 29) met inclusion criteria for Australian school-based wellbeing interventions. All 29 studies scored between 4 and 10 (out of 12) corresponding to low, medium, and high study quality.

### 3.1. Intervention Characteristics

The 29 studies comprised a total sample size of *n* = 13,537 participants. Individual studies varied from 44 participants [57] to 3630 [58]. Fifteen interventions (*n* = 52% of total number of participants) were randomized controlled trials (RCTs) or cluster RCTs, two were non-randomized (*n* = 3%), and 12 (*n* = 45%) were quasi-experimental designs. Students’ age ranged from 5 to 18 years, school grades 1–12, from both metropolitan and rural schools (Table 2). Emergent studies in this field sought to engage a whole of school and community approach that involves parents to actively partake in interventions for children with mental health issues [58,59]. (Table 2)

#### 3.1.1. Study Quality

Out of 29 interventions that measured wellbeing, 2 interventions comprising 3% of the total sample size had high study quality scores. One intervention was a social skill building program [60] that reported no significant effect on wellbeing outcomes. However, a martial arts-based program [73,74] reported small effects on self-efficacy (F(2, 238) = 14.94, *p* < 0.001) but not significant improvements in measures of wellbeing. Both interventions were RCTs that had high exposure of 10–16 weeks and included 3-months follow-up.

Over half of the studies had medium quality scores (*N* = 15, *n* = 78% of the total sample). Within the medium range, 11 were RCTs and 4 were quasi-experimental designs. Eleven studies reported no significant effect on wellbeing. Interventions with low study quality scores (*N* = 12, *n* = 19% of the total number of participants) lacked blinding of participants and/or delivery personnel, were not randomized, had uneven exposure within clusters and control, or no control group, and used one measure (self-report) rather than having measures objectively verified using a range of instruments and assessors (parent, teacher, and clinician). Low study quality interventions were dominated by quasi-experimental designs (*N* = 12, *n* = 16%) compared to RCTs (*N* = 3, *n* = 3%).

#### 3.1.2. Intervention Effect

Seventeen of 29 interventions (*n* = 80% of the total number of participants) reported no statistically significant effect overall on measures related to wellbeing as well as in other related outcomes measures (Table 3). Within the group who reported no significant effect, 11 were RCTs or CRCTs (*n* = 47% of total number of participants) and six were quasi-experimental designs (*n* = 33%).

Eight of 29 interventions reported a significant small effect on outcomes measures, but only three reported an effect on wellbeing outcomes. All three were quasi-experimental designs and involved acceptance and commitment therapy (ACT) (FS *d* = 0.20, *p* = 0.57) [65], psychoeducation (GSE *d* = 0.314, *p* < 0.01) [72], and resilience-building in students with unhealthy perfectionism (CINSS np2 = 0.11, *p* < 0.001) [83]. Two of the interventions had low study scores, while the other had a low–medium study score due to absence of blinding, sampling, randomization, and attrition.

Three interventions reported a statistically significant medium intervention effect, but only one reported on wellbeing outcomes. An intervention based on ACT reported a significant medium effect on flourishing (FS *d* = 0.47, *p* = 0.030) [84]. However, in the absence of data collected for the control group, the outcome measures may not necessarily have achieved significance.

One intervention reported a large effect on social and emotional wellbeing (SEW ŋp2 = 0.16, *p* < 0.01) [61]. This intervention was based on building social–emotional development, well-being, and academic achievement. However, the sample was small, and the participants came from one school.

#### 3.1.3. Intervention Duration and Follow-Up

Intervention duration varied across interventions from 3 weeks [83] to seven days in outdoor camp [85]. The majority (*N* = 21, *n* = 66%) of interventions lasted between six to ten weeks. The two interventions that showed the highest effect ran for 10 weeks [61], and the other had an uneven exposure across clusters. There was no significant correlation between the duration of exposure and study effect.

Eighteen out of 29 interventions had a follow-up which varied from eight-weeks [72] to 24 months [58]. There was no correlation between the period or number of follow-ups and study effect.

#### 3.1.4. Delivery Mode

The delivery mode varied across interventions. Fourteen interventions (*N* = 14, *n* = 78%) were delivered by the schoolteacher or staff (with and without training), including the school nurse and school psychologist. Program staff delivered 25 interventions (*N* = 25, *n* = 32%) and included psychologists, student psychologists, and researchers. In some publications, it was unclear whether the psychologist was a school counsellor or an external psychologist belonging to project staff. It is assumed that the allocation sequence was adequately generated in all studies, unless it was an interrupted series [76], or in circumstances where the school chose the intervention and control groups [85] rather than adhering to a blinded randomization process.

### 3.2. Wellbeing Outcomes

Five different types of wellbeing were identified corresponding to measures of general wellbeing, emotional wellbeing, psychosocial wellbeing, social wellbeing, and subjective wellbeing. Wellbeing outcomes are also measured by other terms directly related to wellbeing that include *eudaimonic* forms, such as flourishing, resilience, life satisfaction, and quality of life. Wellbeing measures also include an individual’s capacity to recover from adverse events and these are measured in terms of protective factors, coping styles, self-esteem, and self-efficacy. Table 4 summarizes the wellbeing outcome(s) that each intervention targeted, and the measuring instrument used to measure each outcome.

#### 3.2.1. Wellbeing

Out of 29 school-based interventions, 8 interventions measured wellbeing through validated measuring instruments (CA, FS, RWBS, SWEM, WEMWBS) including wellbeing-risk (K10) [57]. Out of 8, one intervention reported a significant medium effect on wellbeing and was related to music (WEMWBS. *d* = 0.26, *p* < 0.08) [71]. Two intervention measured emotional wellbeing and involved a whole school approach (MHI *d* = −0.24, *p* = 0.02) [58] and two a music intervention (El Sistema [76], but both reported no significant impact on emotional wellbeing outcomes. Four interventions measured subjective wellbeing and involved ACT [65], resistance training [80], and two involved positive psychology [64,67]. Only ACT [65] as an intervention showed a small effect on subjective wellbeing measures (FS *d* = 0.20, *p* = 0.57). One music-based intervention measured psychosocial wellbeing [76] and was based on El-Sistema-inspired music for largely disadvantaged groups. It measured three areas of wellbeing as well as protective factors and reported a large effect on social wellbeing outcomes (CA *d* = 0.28, *p* = 0.06).

#### 3.2.2. Flourishing

Flourishing is also a measure of wellbeing. A small effect on flourishing was evident using an online positive psychology intervention (SWEMWBS *d* = 0.26, *p* = 0.02) [63], and a medium effect was found for an intervention that combined ACT with self-determination theory (FS *d* = 0.47, *p* = 0.030 [84].

#### 3.2.3. Resilience

Resilience was measured in 7 out of 29 wellbeing interventions using several instruments (CD-RISK, CYRM, Kidscope, RS, RYDM, SDQ, SEARS). Two interventions involved outdoor related activities, namely, football [73,74,75] and wellbeing warriors [73,74] but reported no significant effect on resilience outcomes. A third intervention using behaviour activation and emotional regulation techniques [70], and a fourth study focusing on self-efficacy, resilience, and coping strategies [72] both reported no significant effects on resilience outcomes. One intervention based on psychoeducation [82] reported small effects on competence, relatedness, and autonomy, but not on resilience or wellbeing. A comprehensive intervention based on social and emotional skills-building focused on the areas of emotional literacy, personal strengths, positive coping strategies, problem-solving strategies, stress management and emotional regulation, help-seeking with peer support [57]. In this study, resilience was measured in five areas (Resilience Internal Assets (RYDM); Resilience School Resources 1 (RYDM); Resilience School Resources 2 (RYDM); Resilience Cooperation and Communication (RYDM); Resilience Class Connectedness (RYDM)). This study reported no significant intervention effect on any of measures.

#### 3.2.4. Quality of Life

Quality of life and life satisfaction measures (MSLSS, PedQl 4.0TM, SLSS) are associated with wellbeing. Two interventions that used social skills building techniques [60,78], and a third that reduced screen time using self-determination theory [62], each reported no significant effects on quality of life or life satisfaction. However, a friendship building skills program for depression reported a small but significant effect on life satisfaction (MSLSS *d* = 0.2, *p* < 0.01) [78].

#### 3.2.5. Self-Esteem

Self-esteem was associated with wellbeing outcomes and measured using PSDQ, CSFEI, and RSES. Two interventions based on music therapy [77] and resistance training [80], respectively, reported no effect on self-esteem. Two psychoeducation-based interventions, one based on emotional freedom techniques [81] and CBT-based health and education module [82] also reported no effect on self-esteem.

#### 3.2.6. Self-Efficacy

Self-efficacy has recently been associated with wellbeing [6] and was measured in three interventions using CYRM, GSE, and SES. Two interventions reported a small effect, one based on martial arts (F(2, 238) = 12.14, *p* < 0.001) [73,74] and the other based on building resilience in in regional youth (GSE *d* = 0.314, *p* < 0.01) [72]. A third intervention based on an outdoor youth program reported a significant medium-to-large effect on self-efficacy (F = 20.38, *p* < 0.001) [79].

#### 3.2.7. Protective Factors and Coping Styles

Both protective factors and coping style impact on the ability of an individual to sustain wellbeing, and three interventions reported measures (CA, Kidscope, RYDM) in these areas. One was based on music therapy [76] and reported no significant effect on wellbeing. Another intervention involved self-care techniques related to overcoming obstacles, media, and mastery over life, which also reported no significant effect on wellbeing [72]. A third intervention directly targeted protective factors using school-based health promotion, but also reported no significant effect on wellbeing outcomes [31].

## 4. Discussion

The purpose of this systematic review was to examine the effect of intervention on the wellbeing of young people and adolescents in primary and secondary educational context. Focus was given to Australia as its situation in unique in that, despite the high number of government initiatives that support wellbeing, nationally, there has been a 50% decline of wellbeing and other mental health measures since 2007 [86] as well as high relapse rates [87]. Further, a critical review of previous global systematic reviews conducted during the same search period excluded many Australian school-based interventions, although the study with the largest analysis of Australian cases included 10 interventions during the same search period.

Our study identified 29 school-based interventions that measured wellbeing outcomes in a total of 13,537 participants. The main findings of this systematic review are that 18 interventions, comprising 80% of wellbeing measures, found that there was no intervention effect, regardless of the type of intervention implemented. Eight interventions (*n* = 15%) reported a small effect, and three interventions (*n* = 7%) reported a medium size effect on wellbeing outcomes. One intervention reported a large effect on mental health outcomes. This outcome is consistent with other systematic reviews that reported low outcome measures showing largely small effects for universal school-based preventative interventions [32,44,50]. This review supports the claim that large universal prevention interventions with a small effect can produce meaningful improvements at population levels [41,88].

The study quality of interventions was generally low to medium, with only two interventions achieving high study quality scores, and both were RCTs. One intervention was a social skills-building program and the other was a martial arts-based program. The latter reported small effects on self-efficacy but not significant improvements in measures of wellbeing. Fifteen interventions had medium study quality scores and 12 studies had low study quality. As previous research has shown, however, school-based interventions are challenged on many levels [42,50], predominantly in achieving blinding for participants and implementation personnel, and where the study is based only on children’s self-report. Another reason for lower study quality scores is inadequate generation of sequence, including removing control groups post intervention, overlapping of intervention and waitlist control groups, or where schools were allowed to choose their intervention curriculum topics, including duration. Therefore, reported high intervention effects need to be weighed against study quality. This review found a general association between reported high study impact with generally low study quality scores, which supports the findings of international reviews of school-based interventions [36].

Interventions that showed the highest effects on wellbeing were mixed in type. The highest effect on wellbeing was a social and emotional wellbeing program that combined social and emotional development with academic achievement. Two interventions reported small to medium effects on wellbeing outcomes, and these were based on ACT and psychoeducation. Therefore, an effective implementation strategy to combine wellbeing intervention with school-based learning emerged in findings in this review for a positive intervention effect. This finding is supported by recent research that shows that programs with long-term positive outcomes may occur by combining mental health literacy [38].

There were insufficient data to report on an association between delivery personnel and intervention effect. However, a recent systematic review shows that teacher-delivered interventions with training and/or professional development are effective for implementation of school-based interventions [89]. In addition, no association was found between intervention duration and effect; however, sustainability and duration are considered beneficial to producing long-term results in students [38,90]. While long term results through prolonged but low exposure and duration did not reveal a beneficial intervention effect, it may be the case that high exposure and duration may have a significant effect on wellbeing intervention effectiveness. Further research is needed, however, to explore if effects last through follow-up assessments.

This review found that few Australian school-based interventions produced a significant effect on wellbeing outcomes as measured through validated measuring instruments. Music-based interventions and, to a lesser extent, ACT-based interventions reported significant small-to-medium effects on wellbeing outcomes. Flourishing measures had the greatest impact from ACT and self-determination theory-based interventions. A small significant effect on life-satisfaction was reported from a web-based positive psychology program. Martial arts and outdoor youth programs reported a significant medium to large effect on self-efficacy. Although resilience is closely associated with wellbeing, no specific interventions reported a significant effect. Likewise, no intervention effects were reported on self-esteem measures.

Part of the rationale for focusing on Australian school-based wellbeing interventions was the lack of Australian studies included in previous systematic reviews. The *Australian Educational Leader* suggests that despite the vast number of programs that measure wellbeing in Australia, programs may be excluded in global systematic reviews because of low study quality, suggesting that “more high-quality program evaluations are needed across Australia” [91] (page 43). Therefore, while the high number of interventions (80%) reported no statistically significant outcomes on wellbeing, this review supports findings in other international reviews, suggesting that program fidelity and rigour are needed in program design across school-based interventions [36]. In addition, we draw attention back to the WHO definition of mental health, which ties mental health to wellbeing. Mental disorders among 16–24-year-olds in Australia went up 50% since 2007 according to a recent ‘National Study of Mental Health and Wellbeing’ [86]. Post the COVID-19 pandemic, schools are faced with both challenges and opportunities to change the way we approach wellbeing. Placing wellbeing as a primary measure, rather than as a measure that is secondary to a broad range of mental health interventions, may be the opportunity we need to establish wellbeing measures as an effective early detection measure for the onset of major mental health issues.

The implications for school-based personnel are considerable: for teachers who have to address and support students with social and emotional issues in their classroom, these findings indicate that they have limited tools and intervention programs that work. On the other hand, a measure of low wellbeing from a validated measuring instrument may present both an indication of later onset of a more serious mental health issue, and an opportunity for early intervention to break the trajectory leading to full disorder. Learning to measure wellbeing outcomes using validated wellbeing instruments requires mental a certain level of mental health training. School healthcare staff may also require training to understand which implementation criteria produce more favourable outcomes for students. Finally, there are also implications for families. As previous research has shown [91], family involvement with schools tends to produce better outcomes for young people. With the minimal impact of mental health programs in schools, it may be the case that alternative mechanisms may be needed, such as stronger cooperation between schools and families to find wrap-around pathways of support for young people’s wellbeing.

There were several limitations to this review. First, the search period was 2012–2022 and the last 3 years have been disruptive to schools due to COVID-19 pandemic measures in schools. This event prevented research being conducted in Australian schools and much of the data collection and studies may therefore be representative of pre-pandemic levels of wellbeing. Future studies might focus specifically on post pandemic measures of wellbeing in school-based settings, which are likely to reveal greater mental health and wellbeing needs. Second, one of the outcomes, measures of wellbeing (happiness), could not be analysed because of a lack of studies reporting on happiness measures. Third, there was significant heterogeneity among interventions, which varied in terms of research design, engagement metrics, and research methodologies, including data collection, analysis, and reporting. Due to high heterogeneity, aggregated levels of efficacy using meta-analysis were not feasible. Fourth, statistical calculation of effect was not possible in a small number of studies due to data being unavailable (published or through contact with authors). Subsequently, the effect size calculation may not be the exact value, even though the effect reported for each study (in terms of a significant or not-significant measure) was based on each author’s reported results verbatim. Fifth, many interventions reported data based on self-report or from one source (such as only the child report, or only the parent report) that may be partial to acquiescence resulting in false positives. These measures were not always concurrently verified with teacher, parent, and clinician measures. Sixth, the inclusion criteria were restricted to articles published in peer-reviewed journals, which excludes ongoing programs running in Australian schools that have not published their intervention findings nor have been evaluated. Finally, this review only found one intervention related to Aboriginal children. Further, while some interventions did focus on other minority groups, there were no interventions that focussed specifically on ethnic minority groups. As such, wider search terms may be needed to include a wider set of disadvantaged groups, who are known to experience low wellbeing outcomes.

## 5. Conclusions

Wellbeing is a term that is attached to a range of school-based interventions related to child and adolescent health, mental health, mental disorder, and psychological states. In this review, we aimed to narrow the definition of wellbeing to specific measurable criteria, thereby providing an analysis of wellbeing outcomes in school-based interventions. This systematic review found that most interventions (80%) did not report a statistically significant effect on students’ wellbeing outcomes. Yet, there is an increasing burden on schools to manage the wellbeing of students. Therefore, we suggest that wellbeing be utilized more usefully as an early detection measure for mental health and mental disorders. Rather than a secondary measure that appears in all health and mental-health programs, we suggest that researchers, healthcare workers, and school staff may be able to implement more successful intervention strategies through early detection by targeting wellbeing outcomes as an early intervention and prevention strategy for mental health and mental disorders in children and young people.

## Figures and Tables

**Table 1:** Keyword search strategy.

Key Word	Search String
Wellbeing	“wellbeing” OR “well-being” OR “well being” OR “mental wellbeing” OR “mental well-being” OR “mental well being” OR “subjective wellbeing” OR “subjective well-being” OR “subjective well being” OR “flourish *” OR “eudaimonia”
Clinical diagnosis	“mental health” OR “mental illness” OR “mental disorder” OR “psychiatr*” OR “psycholog*”
Negative emotional states	“social and emotional” OR “psychosocial”
Target group	“child*” OR “adolescen*” OR “school age*” OR “school-age*” OR “schoolchild*” OR “school child*” OR “school-child*” OR “youth” OR “young person” OR “student*” OR “pupil*”
Context	“school*” OR “school-based” OR “school based” OR “whole of school” OR “classroom*”
Country	Australia
Filters	English, Humans, from 1 January 2012 to 1 January 2022

**Table 2:** Participant Characteristics.

Authors	Program (Design)	Age (Sample Size)	Delivery	Exposure	Follow-Up	Quality Score (Tot = 12)
Afsharnejad et al. (2022) [60]	KONTAKT (RCT)	12–17 yrs (90)	Certified KONTAKT^®^ trainers	16 × 90 min session	3 months	High
Ashdown et al. (2012) [61]	You Can Do It! (RCT)	5–7 yrs (57)	Teacher (trained)	10 wks	No follow-up	Medium
Babic et al. (2016) [62]	Switch-Off for Healthy Minds (CRCT)	13–15 yrs (322)	Researcher	Over 6 months	No follow-up	Medium
Burckhardt et al. (2015) [63]	Bite Back (RCT)	13–17 yrs (336)	Online delivery teacher (trained)	6 classes for 4–6 wks	No follow-up	Medium
Burckhardt et al. (2016) [64]	Strong Minds (RCT)	15–18 yrs (267)	Researcher (psychologist)	16 × 30 mins twice a wk over 12 wks	No follow-up	Medium
Burckhardt et al. (2017) [65]	Acceptance and Commitment Therapy (Quasi-experimental)	14–16 yrs (48)	Teacher and Program staff (Psychologist)	25 min/wk for 7 wks	5 months	Low
Calear et al. (2016) [66]	e-couch Anxiety and Worry (CRCT)	12–18 yrs (1767)	Online delivery assisted by school staff	40 mins/wk for 6 wks	6 months	Medium
Colla et al. (2016) [67]	Wellbeing (Quasi-experimental)	12–14 yrs (252)	Psychologist	45 mins/wk for 6 wks	3 months	Medium
Dray et al. (2017) [31]	Pragmatic school-based intervention (CRCT)	12–16 yrs (2149)	School based delivery	16 Strategies of varying hrs	No follow-up	Medium
Gold (2017) [68]	Group Music Therapy (CRCT)	12–14 yrs (89)	Music therapist	Uneven exposure from zero to 62% over 10 wk term	3 months	Medium
Johnson et al. (2016) [69]	Dot be Mindfulness Program (CRCT)	13–14 yrs (258)	External facilitator	35–60 mins/wk for 8 wks	3 months	Low
Johnstone (2020) [70]	Emotion regulation (ERP) and behavioural activation (BAP) programs (CRCT)	8–13 yrs (295)	Project staff (Psychologist)	50 min/wk for 8 wks	6 and 12 months	High
Martin and Wood (2017) [71]	Hoyloake’s DRUMBEAT (Quasi-experimental)	12–14 yrs (61)	Accredited DRUMBEAT facilitator	8 programs/mnth for 7 months	No follow-up	Low
McAllister et al. (2018) [72]	iCARE-R (Quasi-experimental)	13 yrs (850)	Nurses, guidance officers, and teachers	40 mins/wk for 6 wks	2 months	Low
Midford et al. (2017) [57]	Social and Emotional Education (Quasi-experimental)	13–14 yrs (44)	Teacher (trained)	1 wk for 10 wks	No follow-up	Low
Moore et al. (2019, 2021) [73,74]	Well-being warriors	13–14 yrs (283)	Project and school psychologist	50 mins/wk for 10 wks	3 months	High
Nathan et al. 2013 [75]	Football United (Quasi-experimental)	13–17 yrs (142)	Student coaches and school staff	Duration unknown	No follow-up	Low
Osborne (2016) [76]	El-Sistema Inspired Music (Quasi-experimental)	9–11 yrs (128)	School 1 (Melbourne Symphony Orchestra staff). School 2 (School based music staff)	Uneven hrs for 42 months School 1 (Orchestra + Control) and 2 (El-Sistema + Control)	12 months	Low
Rickard et al. (2012) [77]	Kodaly Music Training (CRCT)	6–18 yrs (359)	School staff	Prep/Grade 1: 30 mins/wk singing. Grade 3: 60 mins/wk	No follow-up	Medium
Rose et al. (2014) [78]	Resourceful Adolescent Program (RAP) and Peer Interpersonal Relatedness (PIR) Program (RCT)	9–14 yrs (210)	Clinical psychologist	45 mins/wk for 11 wks (RAP) and 9 wks PIR	5 and 14 months	Medium
Rose et al. (2018) [79]	Outdoor Youth Program (Quasi-experimental)	15–16 yrs (160)	Program delivery staff (*n* = 11)	Uneven exposure School 1: 9-days. School 2: 5–days. School 3: 6 days	3 months	Medium
Smith et al. (2018) [80]	Resistance Training for Teens (CRCT)	9–11 yrs (508)	Teacher delivered	90 mins/wk for 10 wks + 20 min/wk for 5 wks	No follow-up	Medium
Spence et al. (2013) [58]	beyond blue: the national depression initiative (Quasi-experimental)	12–13 yrs (3630)	Teacher delivered	Exposure undefined	24 months	Medium
Stapleton et al. 2017 [81]	Emotional Freedom Techniques (Non-randomised)	13–16 yrs (204)	Project staff (Psychology students)	40 mins/wk for 7 wks	3 and 12 months	Low
Tomyn et al. (2016) [82]	Think Health and Wellbeing (Non-randomised)	13–17 yrs (194)	Trained psychology students	CBT 50 mins/wk for 6 wks	3 months	Low
Vekas et al. (2017) [83]	Minding Young Minds (Quasi-experimental)	10–13 yrs (212)	Research and teacher	CBT 45 mins/wk for 3 wks	3 months	Medium
Vella-Brodrick et al. (2020) [59]	Timbertop (Quasi-experimental)	13–14 yrs (198)	School teachers and staff	1 hr/wk for 1 yr	No follow-up	Low
White et al. (2022) [84]	Health and Well-being for Girls (RCT)	13–14 yrs (89)	Research team	5 hrs/fortnight for 20 wks	No follow-up	Low
Williams et al. (2018) [85]	Outdoor Youth Programs Research Alliance (Quasi-experimental)	14–16 yrs (335)	School, program staff, and outdoor professional	7 days/yr	6 months	Low

**Table 3:** Statistically significant effect of intervention on wellbeing outcomes.

Authors	Program	Intervention Content	Intervention Efficacy (Effect Size)	Study Effect
Afsharnejad et al. (2022) [60]	KONTAKT	Social skills group training for ASD	Quality of life (PedQl 4.0TM-A *d* = 0.18, *p* = 0.40)	No statistically significant effect
Ashdown et al. (2012) [61]	You Can Do It! Early Childhood Education program	SEL for social and emotional development, wellbeing, and academic achievement	Social-emotional well-being (SEW ŋp2 = 0.16, *p* < 0.01)	Significant large effects on social-emotional well-being
Babic et al. (2016) [62]	Switch-Off for Healthy Minds	Reducing screen time using self-determination theory	Quality of life (PedQl 4.0TM-A d not given); Well-being (FS *d* not given)	No statistically significant effect
Burckhardt et al. (2015) [63]	Bite Back RCT	Positive psychology online to improve wellbeing	Life Satisfaction (SLSS) not significant and results not reported; Flourishing (SWEMWBS *d* = 0.26, *p* = 0.02)	Significant small effect
Burckhardt et al. (2016) [64]	Strong Minds	Positive psychology with ACT for dysfunctional cognitive appraisal	Subjective Wellbeing (FS *d* = 0.16, *p* = 0.12)	No statistically significant effect
Burckhardt et al. (2017) [65]	Acceptance and Commitment Therapy (ACT)	Mindfulness for depression and anxiety	Subjective wellbeing (FS *d* = 0.20, *p* = 0.57)	Significant small effect on resilience
Calear et al. (2016) [66]	e-couch Anxiety and Worry	CBT and psychoeducation for anxiety and worry	Wellbeing (WEMWBS *d* = −0.06, *p* = 0.001)	No statistically significant effect
Colla et al. (2016) [67]	Wellbeing	Positive thinking information module and group discussions for subjective wellbeing	Wellbeing (PWI-SC β = 0.01, *p* = 0.996)	No statistically significant effect
Dray et al. (2017) [31]	Pragmatic school-based intervention	Resilience and protective factors for mental health problems	Protective factors Internal (RYDM *d* = 0.02, *p* = 0.81) Protective factors External (RYDM *d* = 0.02, *p* = 0.87)	No statistically significant effect
Gold (2017) [68]	Group Music Therapy	Music therapy for mental health problems	Psychosocial well- being (MHC-SF *d* = 0.12, *p* = 0.706)	No statistically significant effect
Johnson et al. (2016) [69]	Dot be Mindfulness Program	Mindfulness for depression, anxiety and eating disorders	Wellbeing (WEMWBS *d* = 0.09 *p* < 0.001)	No statistically significant effect
Johnstone (2020) [70]	Emotion regulation (ERP) and behavioural activation (BAP)	Resilience to combat worry using emotion regulation and behavioural activation	Resilience (CYRM-12, *d* = 0.06, *p* = 0.92)	No statistically significant effect
Martin and Wood (2017) [71]	Hoyloake’s DRUMBEAT	Music therapy to improve mental health and behaviour problems	Wellbeing (WEMWBS. *d* = 0.26, *p* < 0.08)	No statistically significant effect.
McAllister et al. (2018) [72]	iCARE-R	Self-efficacy, resilience, and coping strategies for mental health problems	Self-efficacy (GSE *d* = 0.314, *p* < 0.01); Social Emotional Assets and Resilience (SEARS-A); Coping styles (Kidcope, Interviews)	Significant small effect in self-efficacy
Midford et al. (2017) [57]	Social and Emotional Education	SEL	Wellbeing Risk (K10 t = 2.00, *p* = 0.053); Resilience Internal Assets (RYDM t = −0.13, *p* = 0.898); Resilience School Resources 1 (RYDM t = −0.97, *p* = 0.337); Resilience School Resources 2 (RYDM t = −1.01, *p* = 0.282); Resilience Cooperation and Communication (RYDM t = −2.34, *p* = 0.024); Resilience Class Connectedness (RYDM t = −2.46, *p* = 0.018); Social and emotional skills (instrument developed for the program t = 0.52, *p* = 0.603)	No statistically significant effect
Moore et al. (2019, 2021) [73,74]	Well-being warriors	Martial arts-based psycho-social training for mental health	Resilience (CYRM F(2, 238) = 18.58, *p* < 0.001); Social self-efficacy (SEQ-C F(2, 238) = 12.14, *p* < 0.001); Emotional self-efficacy (SEQ-C F(2, 238) = 11.64, *p* < 0.001); Total self-efficacy (SEQ-C F(2, 238) = 14.94, *p* < 0.001)	Statistically significant small effect on all measures
Nathan et al. 2013 [75]	Football United	Football to support peer, prosocial, and cross-cultural relationships	Resilience (SDQ *d* = 0.09, *p* = 0.59)	No statistically significant effect
Osborne (2016) [76]	El-Sistema Inspired Music	Music therapy to build emotional wellbeing	School 2. Total well-being (CA) (*d* = 0.09, *p* = 0.08; Emotional well-being (*d* = 0.28, *p* =0.08); Social well-being (*d* = 0.28, *p* = 0.06); Protective factors (*d* = 0.09, *p* = 0.08)	Significant small effect on social and emotional wellbeing
Rickard et al. (2012) [77]	Kodaly Music Training	Music therapy to build social competence and self-esteem	Global self-esteem (CSFEI-3) *F*(1, 91) = 6.38, *p* = 0.013); General self-esteem (*F*(1, 91) = 5.63, *p* = 0.020); Social self-esteem (*F*(1, 77) = 5.24, *p* = 0.025)	Significant small effect for global and general self esteem
Rose et al. (2014) [78]	Resourceful Adolescent Program (RAP) and Peer Interpersonal Relatedness (PIR)	Friendship-building skills for depressive symptoms	Life satisfaction (MSLSS *d* = –0.2, *p* < 0.01)	Significant small effect on life satisfaction
Rose et al. (2018) [79]	Outdoor Youth Program	Outdoor youth program for wellbeing	Self-efficacy (GSES F = 20.38, *p* < 0.001); Wellbeing (RWBS F = 0.0, *p* = 1.00)	Significant medium to large effect for Self-efficacy
Smith et al. (2018) [80]	Resistance Training for Teens	Resistance training for wellbeing	Self-esteem (PSDQ); Subjective well-being (FS β = 0.03, *p* = 0.509)	No effect for regular family support group
Spence et al. (2013) [58]	beyond blue: the national depression initiative	Whole school community for depression prevention	Emotional wellbeing (MHI *d* = −0.24, *p* = 0.02)	No statistically significant effect
Stapleton et al. 2017 [81]	Emotional Freedom Techniques	Emotional freedom techniques for wellbeing	Resilience (CD-RISC *F* (2.68, 31.23) = 0.57, *p* = 0.62)*;* Self-esteem (RSES *F* (3.00, 5.22) = 0.52, *p* = 0.67)	No significant effect on resilience or self-esteem
Tomyn et al. (2016) [82]	Think Health and Wellbeing Non-randomised	Psychoeducation for depression prevention	Resilience (RS χ2 (1, N = 252) = 0.50, *p* = 0.482), Self-esteem (RSES χ2 (1, N = 252) = 1.01, *p* = 0.316)	Significant small effect on social support
Vekas et al. (2017) [83]	Minding Young Minds	Psychoeducation for unhealthy perfectionism	Well-being (MSPSS *d* = 0.28, *p* = 0.046)	Significant small effect wellbeing.
Vella-Brodrick et al. (2020) [59]	Timbertop	Whole of school positive education for wellbeing	Competence CINSS (np2 = 0.09, *p* < 0.001); Relatedness (np2 = 0.09, *p* < 0.001); Autonomy (np2 = 0.11, *p* < 0.001)	Significant small effect on competence, relatedness, autonomy
White et al. (2022) [84]	Health and Well-being for Girls	Self-determination theory and ACT for health and wellbeing	Flourishing Internalizing (FS *d* = 0.47, *p* = 0.030)	Significant medium effect on flourishing internalising
Williams et al. (2018) [85]	Outdoor Youth Programs Research Alliance	Outdoor adventure therapy to promote positive adjustment	Wellbeing (SWEMWBS *d* = 0.03, *p* not given)	No statistically significant effect

**Table 4:** Wellbeing Measures.

Authors	General Wellbeing	Emotional Wellbeing	Psychosocial Wellbeing	Social Wellbeing	Subjective Wellbeing	Coping Styles	Flourishing	Life Satisfaction	Quality of Life	Protective Factors	Resilience	Self- Esteem	Self- Efficacy
Afsharnejad et al. (2022) [60]									PedQl				
Ashdown et al. (2012) [61]				WBS									
Babic et al. (2016) [62]	FS								PedQl				
Burckhardt et al. (2015) [63]							SWEMWBS	SLSS					
Burckhardt et al. (2016) [64]					FS								
Burckhardt et al. (2017) [65]					FS				60				
Calear et al. (2016) [66]	WEMWBS												
Colla et al. (2016) [67]					PWI-SC								
Dray et al. (2017) [31]										RYDM			
Gold (2017) [68]			MHC										
Johnson et al. (2016) [69]	WEMWBS												
Johnstone (2020) [70]											CYRM		
Martin and Wood (2017) [71]	WEMWBS												
McAllister et al. (2018) [72]						Kidscope					SEARS		GSE
Midford et al. (2017) [57]	K10										RYDM		
Moore et al. (2019, 2021) [73,74]											CYRM		CYRM
Nathan et al. 2013 [75]											SDQ		
Osborne (2016) [76]	CA	CA		CA						CA			
Rickard et al. (2012) [77]												CSFEI	
Rose et al. (2014) [78]								MSLSS					
Rose et al. (2018) [79]	RWBS												GSES
Smith et al. (2018) [80]					FS							PSDQ	
Spence et al. (2013) [58]		MHI											
Stapleton et al. 2017 [81]											CD-RISC	RSES	
Tomyn et al. (2016) [82]											RS	RSES	
Vekas et al. (2017) [83]	MSPSS												
Vella-Brodrick et al. (2020) [59]													
White et al. (2022) [84]							FS						
Williams et al. (2018) [85]	SWEMWBS												

CA Clowning Around; CD-RISC Connor–Davidson Resilience Scale; CSFEI Culture-Free Self Esteem Inventory; CYRM Child and Youth Resilience Measure; FS Flourishing Scale; K-10 Kessler-10; GSE General Self-Efficacy Scale; MHC Mental Health Continuum; MHI Mental Health Inventory; MSLSS Multidimensional Students Life Satisfaction Scale; MSPSS Multidimensional Scale of Perceived Social Support; PedQl 4.0TM Pediatric Quality of Life Inventory TM; PSDQ Physical Self Description Questionnaire; PWI-SC Personal Well-being Index—School Children; RS Resilience Scale; RSES Response to Stressful Experiences Scale; RWBS Religious well-being scale; RYDM Resilience and Youth Development Module; SEARS Social Emotional Assets and Resilience Scale; GSES Generalized Self Efficacy Scale; SDQ Strengths and Difficulties Questionnaire; SLSS Life Satisfaction Scale; SWEMWBS Short Warwick Edinburgh Mental Wellbeing Scale; WBS Wellbeing Scale; WEMWBS Warwick Edinburgh Mental Wellbeing Scale.

## Data Availability

The datasets used and/or analysed during the current study are available from the corresponding author upon reasonable request.
